# Early Antimicrobial De-escalation and Stewardship in Adult Hematopoietic Stem Cell Transplantation Recipients: Retrospective Review

**DOI:** 10.1093/ofid/ofx226

**Published:** 2017-12-11

**Authors:** Matthew Snyder, Yanina Pasikhova, Aliyah Baluch

**Affiliations:** 1 Department of Pharmacy, H. Lee Moffitt Cancer Center and Research Institute, Tampa, Florida; 2 Department of Infectious Diseases, H. Lee Moffitt Cancer Center and Research Institute, Tampa, Florida

**Keywords:** antimicrobial de-escalation, antimicrobial stewardship, broad-spectrum antimicrobials, hematopoietic stem cell transplant, neutropenic fever

## Abstract

**Background:**

Antimicrobial stewardship in allogeneic hematopoietic stem cell transplantation (allo-HSCT) recipients remains underutilized in North America. European guidelines advise de-escalation of broad-spectrum therapy after 72 hours in select patients with neutropenic fever of unknown origin. This is not commonplace in the United States, as current guidelines recommend broad-spectrum therapy until neutrophil engraftment. If de-escalating after at least 5 days of broad-spectrum therapy and defervescence in neutropenic allo-HSCT recipients does not predispose them to recurrent fever or infection, the practice could afford several benefits.

**Methods:**

The primary end point was rate of recurrent fever. Secondary outcomes included *Clostridium difficile*–associated infections, length of stay, intensive care unit (ICU) admission incidence, in-hospital mortality rate, need for re-escalation of therapy, rate of positive blood cultures for patients who had recurrent fevers, overall antimicrobial utilization from neutropenic fever onset, and pharmacoeconomic impact.

**Results:**

A total of 120 patients were assessed in 2 groups as cohort 1 (n = 46), which received early de-escalation, and cohort 2 (n = 74), which did not. The primary end point met criteria for noninferiority, as 7 patients (15%) in cohort 1 had recurrent fever within the specified time frame compared with 14 (19%) in cohort 2 (90% CI, –0.0878 to 0.1629, *P* = .026). Patients in cohort 1 received significantly less gram-positive broad-spectrum antimicrobials, with trends toward lower use of broad-spectrum gram-negative agents and lower associated costs and no differences in length of stay, ICU admission incidence, need for re-escalation of therapy, rate of culture-positive bacteremia after de-escalation or discontinuation of broad-spectrum therapy, or in-hospital mortality rate.

**Conclusions:**

De-escalating after at least 5 days of broad-spectrum therapy and defervescence did not appear to affect the rate of recurrent fever. This allowed for significant reductions in gram-positive broad-spectrum antimicrobial utilization, with trends toward lower use of broad-spectrum gram-negative agents and associated costs and no difference in clinical outcomes compared with those continuing such therapy until neutrophil engraftment.

Antimicrobial stewardship is a vital component of health care at numerous institutions worldwide and affords several well-documented benefits [1–4]. It is widely supported by prominent organizations, including the Infectious Diseases Society of America (IDSA), Centers for Disease Control and Prevention (CDC), and World Health Organization (WHO) [5–7]. While antimicrobial stewardship practices have been extensively evaluated in general areas of medicine such as the inpatient ward and intensive care unit (ICU), current literature affords far less data in more specialized patient populations. This absence is evident in several fields of oncology, especially allogeneic hematopoietic stem cell transplantation (allo-HSCT).

While prophylactic antimicrobial strategies for patients undergoing allo-HSCT vary by practice, the majority employ the use of an antipseudomonal fluoroquinolone or third-generation cephalosporin, an antifungal, and an antiviral agent. At our institution, ciprofloxacin is the drug of choice for antibacterial prophylaxis and is substituted by levofloxacin if there is a need for improved streptococcal coverage. Cefdinir is only used when fluoroquinolones are contraindicated (QT prolongation, allergy, etc.). In addition, patients receive antiviral prophylaxis with acyclovir and antifungal prophylaxis, along with the antibacterial agent starting on the day of stem cell infusion or onset of neutropenia, whichever occurs first. While patients will remain on prophylactic agents in the immediate post-transplant period, the majority will develop a neutropenic fever (NPF) at some point in the following weeks. Given their expected prolonged and profound neutropenia post-transplant, allo-HSCT recipients are classified as high risk by the current IDSA and National Comprehensive Cancer Network (NCCN) guidelines [8, 9]. These patients should be escalated to empiric broad-spectrum coverage at the onset of neutropenic fever, with subsequent therapy directed toward culture and susceptibility results. However, a pathogen is only identified in 20%–30% of cases, leaving most patients with no identifiable source of fever. In such patients with neutropenic fever of unknown origin, these guidelines recommend the administration of broad-spectrum antimicrobials until a consistently increasing absolute neutrophil count (ANC) of more than 500 cells/mm^3^ is observed at least once and no fever is present for more than 48 hours, regardless of the patient’s clinical status or true validity of an infectious etiology [8, 9]. Despite this recommendation, some literature suggests that this ANC cutoff value is of limited utility in determining treatment duration or patient discharge and may not be necessary [10, 11].

Given how infrequently an infectious source is identified in neutropenic fever episodes, practicing antimicrobial stewardship in this setting is equally if not more important than in the general patient population [8, 12]. Excessive broad-spectrum antimicrobial therapy during the prolonged neutropenia allo-HSCT recipients’ experience can promote the selection of multidrug-resistant (MDR) pathogens [13]. It is imperative to avoid further contribution to this problem as many HSCT recipients already possess several risk factors for developing infection with resistant bacteria (prior colonization or infection by resistant pathogens, previous exposure to broad-spectrum therapy [including third-generation cephalosporins], and prolonged and frequent hospital stays) before ever undergoing transplantation [14]. Unwarranted continued use of broad-spectrum agents further increases patients’ predisposition to infection by fungi and *Clostridium difficile*, both of which greatly contribute to morbidity and mortality in HSCT recipients [15–18].

Growing antimicrobial resistance in patients with hematologic malignancies and HSCT recipients has been recognized by countries around the world, prompting some organizations to move away from widely accepted practices and endorse more aggressive de-escalation of antimicrobial therapy when treating high-risk patients with neutropenic fever. The 2011 4th European Conference on Infections in Leukemia (ECIL) guidelines recommend discontinuation of broad-spectrum antibiotics after 72 hours in neutropenic patients with fevers of unknown origin who have been hemodynamically stable since presentation and afebrile for at least 48 hours, regardless of ANC or expected duration of neutropenia [14]. Although a number of other publications promote similar concepts, this degree of early de-escalation of antimicrobial therapy in the management of neutropenic fever is still not commonplace in general clinical practice [19, 20]. A commonly cited rationale for this is the theoretical concern that stopping broad-spectrum therapy early may increase the potential for recurrent infection and subsequent need for re-escalation of antimicrobials, as cited in an early work by Pizzo and colleagues [12]. However, these concerns have been demonstrated to be unfounded in several subsequent studies [21–23].

Optimizing the use of antimicrobials has also been shown to reduce hospital drug costs [4, 24]. The recent increase of unexpected manufacturer drug shortages that are still ongoing has made the necessity of optimizing the use and conservation of affected antimicrobials, such as piperacillin-tazobactam, ceftazidime, and cefepime, even more evident. Much effort has been put into analyzing these shortages and trying to determine how to mitigate and ultimately prevent them from occurring altogether [25]. A considerable interest remains in promoting the early de-escalation of antimicrobial therapy to avoid the detrimental effects and costs associated with the prolonged, unnecessary use of these agents in HSCT recipients. The purpose of this study was to retrospectively compare early antimicrobial de-escalation in allo-HSCT recipients with continuation of broad-spectrum therapy until neutrophil engraftment.

## METHODS

This study was approved by the Institutional Review Board of the University of South Florida and the H. Lee Moffitt Cancer Center and Research Institute Scientific Review Committee and consisted of a retrospective review of patients who received an allo-HSCT from January 1, 2014, to December 31, 2015, at our tertiary care cancer institution.

Patients’ clinical data were retrieved from the institution’s electronic medical record system. Patients ≥18 years of age who received an allo-HSCT and developed neutropenic fever (ANC < 500 cells/mm^3^ or expected ANC < 500 cells/mm^3^ over the next 48 hours in combination with a single oral temperature ≥38.3°C [101°F] or 38°C [100.4°F] sustained over 1 hour [8]) of unknown origin were included in either cohort 1 or cohort 2 based on time of discontinuation of broad-spectrum antimicrobial therapy. Escalation of antimicrobial therapy occurred at onset of neutropenic fever and was defined as switching from a prophylactic antibacterial agent (levofloxacin, ciprofloxacin, cefdinir) to broad-spectrum therapy: an antipseudomonal beta-lactam (aztreonam, cefepime, ceftazidime, meropenem, piperacillin-tazobactam), alone or in combination with an agent with expanded gram-positive coverage (vancomycin, daptomycin, and linezolid) and/or an aminoglycoside (tobramycin). Per institutional protocol, a gram-positive agent is recommended empirically only in patients who are colonized with a resistant gram-positive organism (methicillin-resistant *Staphylococcus aureus* [MRSA], vancomycin-resistant *Enterococcus* [VRE], or penicillin-resistant *Streptococcus pneumoniae*), those with hemodynamic instability, clinically suspected serious catheter-related infection, skin or soft tissue infection at any site, radiographically documented pneumonia in a setting of MRSA colonization and severe mucositis if fluoroquinolone prophylaxis has been given and ceftazidime is employed as empirical therapy. Furthermore, empiric addition of tobramycin is reserved for patients with history of infection or colonization with an MDR gram-negative pathogen, those who exhibit signs of septic shock, or patients who are otherwise clinically unstable. In the absence of a proven infection, empiric gram-positive agents and tobramycin should be discontinued after 48 hours.

Cohort 1 represents patients who were de-escalated early, defined as switching from broad-spectrum antimicrobial therapy to their original prophylactic antimicrobial agent while still neutropenic. Switches between broad-spectrum agents (eg, meropenem to cefepime) were not included in this cohort. Cohort 2 represents patients who continued such therapy until neutrophil engraftment (ANC ≥ 500 cells/mm^3^ for 3 consecutive days [26]). All patients received a minimum of 5 days of broad-spectrum antimicrobial therapy, had defervesced, and were afebrile at time of antimicrobial de-escalation or discontinuation. Defervescence was defined as the absence of a temperature of ≥38°C [100.4°F] for at least 48 consecutive hours, as recommended by the IDSA and ECIL guidelines [8, 14]. A 5-day minimum duration was selected to correlate with the time required for blood cultures drawn at neutropenic fever onset to finalize. As there was no institutionalized de-escalation protocol at the time of the study, patient selection for de-escalation after meeting the above criteria was based on a recommendation from the Infectious Diseases consult service. However, the final decision to de-escalate remained at the discretion of the primary transplant team, accounting for why some patients were de-escalated early whereas others were not. Patients were excluded if they remained afebrile throughout the period of neutropenia, received any broad-spectrum antimicrobial therapy from day –7 of transplant to the onset of neutropenia (non-neutropenic fever), or had a documented or clinically diagnosed source of infection by culture, imaging, or other means. Subjects receiving haploidentical or cord blood transplants and those undergoing early transition to the outpatient setting less than a week after transplant were also excluded. Methicillin-resistant *Staphylococcus aureus* and vancomycin-resistant *Enterococcus* polymerase chain reaction (PCR) screening was implemented on the day of admission for every patient, with VRE screening conducted on a once weekly basis thereafter. Antimicrobial order and utilization data were captured via bar code administration of medications and the associated documentation of time, route, drug, dose, and dosing frequency in the electronic medical record.

## OUTCOMES AND TRIAL DESIGN

**Figure 1.**
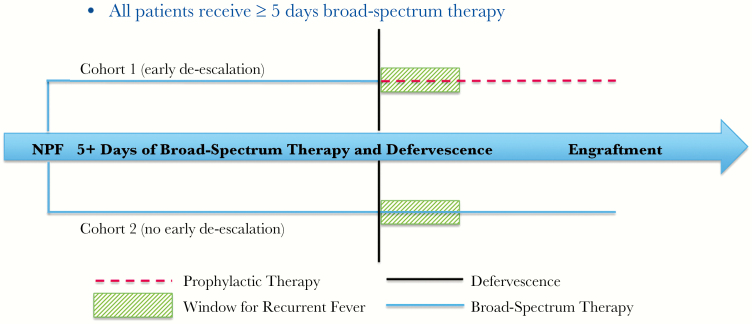
Summary overview of trial design to evaluate early antimicrobial de-escalation in allogeneic hematopoietic stem cell transplantation (allo-HSCT) recipients. After HSCT and development of neutropenic fever, all patients in the study received at least 5 days of broad-spectrum antimicrobial therapy before undergoing assessment for the primary end point, which was the rate of recurrent fever within a 72-hour time frame. This period occurred at a different time point for every individual patient. For those who were de-escalated early (cohort 1), the time frame began when each patient had been afebrile for ≥48 hours and de-escalated from broad-spectrum therapy to his or her original prophylactic agent, prior to neutrophil engraftment. For patients who continued broad-spectrum antimicrobials until neutrophil engraftment (cohort 2), this period started when they had been afebrile for ≥48 hours and would have been eligible for de-escalation. Time frames for patients who defervesced earlier than day 5 of neutropenic fever were not initiated until after the requirement for antimicrobial duration was met. Abbreviation: NPF, neutropenic fever.

The primary end point was rate of recurrent fever within 72 hours of antimicrobial de-escalation. A 72-hour time frame was chosen as it has been previously utilized in an effort to minimize inclusion of any fever or antimicrobial re-escalation secondary to new infectious and/or noninfectious causes [21]. This period occurred at a different time point for every individual patient. For those who were de-escalated early (cohort 1), the time frame began when each patient had been afebrile for ≥48 hours and de-escalated from broad-spectrum therapy to his or her original prophylactic antimicrobial agent, prior to neutrophil engraftment. For patients who continued broad-spectrum antimicrobials until neutrophil engraftment (cohort 2), this period started when they had been afebrile for ≥48 hours and would have been eligible for de-escalation. Timeframes for those who defervesced earlier than day 5 of neutropenic fever were not initiated until the requirement for antimicrobial duration was met (Figure 1). These windows of time were selected to compare similar periods in the neutropenic fever course of both cohorts, allowing us to better assess if continuing broad-spectrum antimicrobial therapy until neutrophil engraftment had an impact on rate of recurrent fever. Secondary analysis was performed on a number of prespecified outcomes, including *Clostridium difficile*–associated infections, length of stay (overall and of survivors only), ICU admission incidence, in-hospital mortality rate, need for re-escalation of therapy (switching from a prophylactic to broad-spectrum antimicrobial agent after the patient’s therapy from the first neutropenic fever episode had already been de-escalated in cohort 1 or restarting broad-spectrum antimicrobial therapy after neutrophil engraftment in cohort 2), rate of culture-positive bacteremia after de-escalation or discontinuation of broad-spectrum therapy, overall antimicrobial utilization from neutropenic fever onset, and pharmacoeconomic impact.

## DATA COLLECTION

Demographic information included gender, age, height, weight, and body surface area. Pretransplant information collected included MRSA or VRE colonization, viral serologies (including cytomegalovirus [CMV] host-donor mismatch), Karnofsky Performance Status (KPS) score, Sorror Comorbidity score, and ANC at admission. Primary malignancy, human leukocyte antigen match, graft-vs-host disease (GVHD) prophylaxis regimen, conditioning regimen (myeloablative vs reduced-intensity), and transplant days (any day from day of transplant to discharge or death) were also reported. Other variables assessed included ANC at neutropenic fever onset, quick Sepsis-related Organ Failure Assessment (qSOFA) score at administration of broad-spectrum therapy for neutropenic fever, time to and duration of neutropenic fever, antimicrobial utilization prior to de-escalation, noninfectious causes of fever or prolonged neutropenia post-transplant, and time to neutrophil engraftment. Days of therapy were assessed as any day a patient received broad-spectrum antimicrobials for the management of the first episode of neutropenic fever. To account for patients potentially receiving more antimicrobials secondary to prolonged hospitalizations, utilization data were standardized per 1000 transplant days, which were defined as the number of days from day of transplant to hospital discharge. The WHO Defined Daily Dose (DDD) Index was employed as a measuring unit for each agent as recommended for drug utilization studies [27]. A slight modification was made to the DDD of antimicrobials to accurately reflect the doses used in this study’s patient population for the treatment of neutropenic fever (Table 3). This adjusted DDD (aDDD) provided a fixed unit of measurement independent of price and dosage form and permitted for more accurate and complete comparison of utilization data between groups.

## POWER AND STATISTICAL CALCULATIONS

To calculate the sample size necessary to meet power, an expected success proportion of 6% was utilized based on the rate of recurrent fever described in previous literature [22]. Assuming this proportion with an allocation ratio of 2:1, a 1-sided significance level of 2.5%, and a noninferiority margin of 10%, we estimated that a sample of 101 patients (34 in cohort 1 and 67 in cohort 2) would have 80% power to demonstrate noninferiority in the rate of recurrent fever. A descriptive statistical analysis was conducted to assess the patients’ demographic characteristics. Median or mean values and ranges are provided for continuous variables, and patient numbers and percentages are shown for categorical variables. For the primary end point, cohorts were compared utilizing the Farrington-Manning method to assess noninferiority. Unpaired *t* tests or Mann-Whitney *U* tests were conducted for continuous variables, while *χ*^2^ tests or the Fisher’s exact test were employed for categorical variables. A *P* value of less than .05 was considered statistically significant. The primary end point was calculated with SAS software, while secondary outcome data and patient characteristics were analyzed using IBM SPSS Statistics version 21.

## PHARMACOECONOMIC ANALYSIS

Antimicrobial utilization data were derived based on the entire treatment course of the first episode of neutropenic fever and the first subsequent course of re-escalation therapy if initiated within 72 hours. The rationale was selected with a similar manner of thought—to eliminate antimicrobial re-escalation secondary to new infectious or noninfectious causes. Using the average wholesale price (AWP) at the time of data analysis, every individual dose administered over the study period was assigned a price and summed together to provide the cost of broad-spectrum antimicrobial therapy.

## RESULTS

One-hundred twenty allo-HSCT recipients met assessment criteria during the study period, with 46 in cohort 1 and 74 in cohort 2. Baseline patient characteristics were similar between the 2 groups, as were transplant demographics (Table 1). The majority of patients were male (59% in cohort 1 and 51% in cohort 2), received a myeloablative conditioning regimen (74% and 77%), had a matched unrelated donor (54% and 62%), got a GVHD prevention regimen containing tacrolimus and sirolimus (63% and 74%), and used ciprofloxacin as antimicrobial prophylaxis (74% and 70%). The median ANC at presentation was similar for both cohorts (3 k/uL for cohort 1 and 2.9 k/uL for cohort 2, none in either with profound neutropenia) as all patients were admitted for a scheduled allo-HSCT prior to receiving conditioning chemotherapy (Table 1). Median ANC at the time of NPF was also similar, with values of 0.3 k/uL and 0.2 k/uL in cohorts 1 and 2, respectively (Table 2). There were no differences between the cohorts in baseline performance status (Table 1) or the gram-negative agents used for the initial treatment of neutropenic fever (Table 2). However, despite similar rates of colonization with MRSA or VRE and hemodynamic instability, as assessed by the qSOFA score, at the time of neutropenic fever, the use of empiric gram-positive therapy differed among the groups (Table 2). Seven patients (16%) in cohort 1 received an empiric gram-positive agent compared with 25 patients (33%) in cohort 2 (*P* = .025). Patients in cohort 1 took an average of 18 days to reach neutrophil engraftment (range, 13–28 days), compared with 15 days (range, 11–23 days) in cohort 2 (*P* < .001).

**Table 1.** Baseline Characteristics and Transplant and Disease Demographics and Adjusted Defined Daily Dose Calculations

Variable	Cohort 1 (Early De-escalation) (n = 46)	Cohort 2 (No Early De-escalation) (n = 74)	*P* Value
Gender, n (%)			
Male	27 (59)	38 (51)	.432
Measurables^a^			
Age, y	58 (31–74)	57 (21–72)	.220
Height, cm	173 (157–193)	170 (147–194)	.176
Weight, kg	81 (46–129)	78 (49–152)	.365
BSA, kg/m^2^	1.97 (1.42–2.46)	1.91 (1.46–2.76)	.442
Pretransplant screenings and serologies, n (%)			
MRSA PCR (+)	0 (0)	0 (0)	- - -
VRE PCR (+)	2 (4)	7 (9)	.480
Cytomegalovirus (+)	29 (63)	49 (66)	.844
Hepatitis B virus (+)	1 (2)	0 (0)	.383
Hepatitis C virus (+)	0 (0)	0 (0)	- - -
Varicella zoster virus (+)	39 (85)	65 (88)	.783
Epstein Barr virus (+)	44 (96)	72 (97)	.637
Herpes simplex virus (+)	36 (78)	67 (91)	.104
Human immunodeficiency virus (+)	1 (2)	0 (0)	.383
Laboratory values at admission^a^			
ANC, k/uL	3 (0.3–18.3)	2.9 (0.2–9)	.816
Pretransplant assessment scores^b^			
Karnofsky Performance Status score	91.52 (70–100)	90.68 (70–100)	.552
Sorror Comorbidity score	2.71 (0–8)	2.69 (0–8)	.937
Malignancy			
Acute myeloid leukemia	14 (30)	36 (49)	.108
Myelodysplastic syndrome	12 (26)	9 (12)	
Acute lymphocytic leukemia	5 (11)	9 (12)	
Chronic lymphocytic leukemia	5 (11)	2 (3)	
Non-Hodgkin lymphoma	4 (9)	9 (12)	
Other^c^	6 (13)	9 (12)	
HLA match			
Matched related donor	14 (31)	24 (33)	.191
Matched unrelated donor	25 (54)	46 (62)	
Mismatched unrelated donor	7 (15)	4 (5)	
Conditioning regimen			
Myeloablative conditioning regimen	34 (74)	57 (77)	.698
Reduced-intensity conditioning regimen	12 (26)	17 (23)	
GVHD prophylaxis			
Tacrolimus/sirolimus	29 (63)	55 (74)	.464
Tacrolimus/sirolimus/antithymocyte globulin	4 (9)	4 (5)	
Tacrolimus/sirolimus/interleukin-2	6 (13)	5 (7)	
Tacrolimus/methotrexate	2 (4)	4 (5)	
Tacrolimus/MMF	2 (4)	2 (3)	
Tacrolimus/methotrexate/antithymocyte globulin	1 (2)	0 (0)	
Sirolimus/MMF/post-transplant cyclophosphamide	1 (2)	0 (0)	
Sirolimus/post-transplant cyclophosphamide	1 (2)	1 (1)	
Tacrolimus/sirolimus/ustekinumab	0 (0)	3 (4)	
Antimicrobial prophylaxis			
Ciprofloxacin	34 (74)	52 (70)	.067
Levofloxacin	10 (22)	11 (15)	
Cefdinir	1 (2)	11 (15)	
Overall	45 (98)^c^	74 (100)	.383

Median or mean values and ranges are provided for continuous variables. Characteristics and demographics were assessed via a descriptive statistical analysis. Patient numbers and percentages are shown for categorical variables. Unpaired *t* tests or Mann-Whitney *U* tests were conducted for continuous variables, while χ^2^ tests or the Fisher’s exact test were employed for categorical variables. *P* < .05 was considered statistically significant.

Abbreviations: ANC, absolute neutrophil count; BSA, body surface area; GVHD, graft-vs-host disease; HLA, human leukocyte antigen; MMF, mycophenolate mofetil; MRSA, methicillin-resistant *Staphylococcus aureus*; PCR, polymerase chain reaction; VRE, vancomycin-resistant *Enterococcus*.

^a^Value reported as median (range).

^b^Value reported as mean (range).

^c^Other includes multiple myeloma, acute biphenotypic leukemia, prolymphocytic leukemia, chronic myeloid leukemia, Hodgkin lymphoma, and myelofibrosis.

**Table 2.** Neutropenic Fever and Hospitalization Course

Variable	Cohort 1 (Early De-escalation) (n = 46)	Cohort 2 (No Early De-escalation) (n = 74)	*P* Value
Initial treatment for NPF			
Cefepime	38 (83)	60 (81)	.427
+ tobramycin^a^	0 (0)	1 (1)	
Piperacillin-tazobactam	8 (17)	10 (14)	
Meropenem	0 (0)	3 (4)	
Overall	46 (100)	74 (100)	- - - -
Initial addition of gram-positive agent for NPF			
Vancomycin	3 (7)	12 (16)	.152
Daptomycin	4 (9)	12 (16)	
Linezolid	0 (0)	1 (1)	
Overall	7 (16)	25 (33)	.025
Neutropenia and ANC recovery, d^b^			
ANC at NPF onset, k/uL	0.3 (0–3.3)	0.2 (0–3.4)	.141
Time to first NPF	10 (–1 to 16)	10 (1–16)	.335
Duration of NPF	2 (1–10)	3 (1–17)	.002
Time to neutrophil engraftment	18 (13–28)	15 (11–23)	<.001
Acute assessment score^c^			
qSOFA at administration of broad-spectrum therapy	0.304 (0–2)	0.324 (0–2)	0.837
Hospitalization course			
Recurrent fever within 72-hour time frame, n (%)^d^	7 (15)	14 (19)	.026
Length of stay, d^b^	20 (15–35)	20 (14–49)	.668
Among survivors^e^	20 (15–35)	20 (14–34)	.949
ICU admission, n (%)	0 (0)	2 (3)	.523
*Clostridium difficile*–associated infections, n (%)^f^	2 (4)	1 (1)	.558
Mortality, n (%)	0 (0)	3 (4)	.285
Other etiologies of fever or prolonged neutropenia, n (%)			
Granulocyte-colony stimulating factor	3 (7)	3 (4)	.674
Antithymocyte globulin	5 (11)	4 (5)	.302
Cyclophosphamide	2 (4)	1 (1)	.558
Interleukin-2	6 (13)	5 (7)	.331

Median values and ranges are provided for continuous variables, and patient numbers and percentages are shown for categorical variables. Unpaired *t* tests or Mann-Whitney *U* tests were conducted for continuous variables, while χ^2^ tests and the Fisher’s exact test were employed for categorical variables. *P* < 0.05 was considered statistically significant.

Abbreviations: ANC, absolute neutrophil count; ICU, intensive care unit; NPF, neutropenic fever; qSOFA, quick Sepsis-related Organ Failure Assessment.

^a^One patient received cefepime and tobramycin concomitantly at onset of NPF.

^b^Values are reported as median (range).

^c^Values are reported as mean (range).

^d^The Farrington-Manning method was utilized to assess noninferiority: (90% CI, –0.0878 to 0.1629)

^e^Three patients in cohort 2 died during the hospitalization and were excluded from the comparison to remove any potential bias from this measure.

^f^After broad-spectrum therapy initiation.

For the primary end point, a total of 21 patients in the overall study population (18%) had recurrent fever within the 72-hour time frame. Cohort 1 had 7 such incidences (cohort rate = 15%), while cohort 2 had 14 (cohort rate = 19%), which met criteria for noninferiority (90% CI, –0.0878 to 0.1629, *P* = .026). Re-escalation or re-initiation of broad-spectrum therapy, as previously defined, occurred in 19 of the 21 patients with recurrent fever within 72 hours. Analysis of individual group rates revealed 7 patients in cohort 1 (15%) compared with 12 (16%) in cohort 2 (*P* = .884). In this patient subset, there was no difference in average time to re-escalation (37 hours vs 32 hours, *P* = .837), nor was there a difference in mean days of gram-positive (0.86 vs 1.33, *P* = .918), gram-negative (5.29 vs 4.08, *P* = .422), or overall (6.14 vs 5.41, *P* = .837) agent utilization between cohorts 1 and 2, respectively.

Overall antibiotic use, whether assessed by days of therapy, transplant days, or aDDDs, was less in cohort 1 than cohort 2 for gram-negative agents, carbapenems, and gram-positive agents, with a statistically significant reduction in the latter (Table 3). In cohort 1, a total of 147 days of broad-spectrum antimicrobial therapy were spared due to early de-escalation, an average of 3.2 days per patient. This savings is reflected in cost of broad-spectrum antimicrobials, which was lower in cohort 1 when standardized per patient (*P* = .005) and per 1000 transplant days (*P* = .012) (Table 3). Whether examining length of stay overall (median 20 vs 20 days, *P* = .668) or among survivors only (median 20 vs 20 days, *P* = .949), incidence of ICU admission (0% vs 3%, *P* = .523), occurrence of *Clostridium difficile*–associated infections (4% vs 1%, *P* = .558), or in-hospital mortality rate (0% vs 4%, *P* = .285), there were no significant differences between cohort 1 and cohort 2 in any secondary clinical outcomes for the hospitalization course (Table 2). No patient in either group experienced a culture-positive bacteremia within the specified time frame after broad-spectrum therapy was de-escalated or discontinued.

**Table 3.** Antimicrobial Therapy Selection and Use

Adjusted Daily Defined Dose Calculations				
Drug	Current WHO DDD	NPF Dosing		NPF aDDD
Cefepime	2	2 g every 8 h		6
Ceftazidime	4	1 g every 6 h		4
Aztreonam	4	1 g every 6 h		4
Piperacillin-tazobactam^a^	14	3.375 g every 6 h		14
Meropenem	2	0.5 g every 6 h		2
Tobramycin^b^	0.24	0.35 g (5 mg/kg) every 24 h		0.35
Vancomycin	2	1 g every 12 h		2
Daptomycin^b^	0.28	0.56 g (8 mg/kg) every 24 h		0.56
Linezolid	1.2	0.6 g every 12 h		1.2
Variable		Cohort 1 (Early De-escalation) (n = 46)n (%)	Cohort 2 (No Early De-escalation) (n = 74)n (%)	*P* Value
Days of antimicrobial use per patient, d^c^				
Gram-positive agent		0.6 (0–10)	1.7 (0–11)	.001
Gram-negative agent		7.8 (5–18)	8.4 (5–29)	.534
Carbapenem		0.7 (0–8)	1.3 (0–18)	.522
Overall (gram-positive + gram-negative)		8.3 (5–23)	10.1 (5–36)	.028
Transplant days^d,e^		936	1572	- - -
Per patient		20 (15–35)	20 (14–49)	.668
Days of antimicrobial use per 1000 transplant days^c,e^				
Gram-positive agent		30 (0–500)	78 (0–355)	< .001
Gram-negative agent		389 (172–789)	398 (192–1000)	.668
Carbapenem		38 (0–444)	50 (0–562)	.520
Overall (gram-positive + gram-negative)		416 (172–1150)	477 (192–1105)	.043
Adjusted defined daily doses, aDDD^c^				
Gram-positive agent		31 (0–441)	90 (0–555)	.001
Gram-negative agent		313 (125–737)	324 (120–833)	.401
Carbapenem		33 (0–403)	39 (0–517)	.562
Overall (gram-positive + gram-negative)		344 (125–1100)	414 (120–970)	.021
Antimicrobial cost, $^c^				
Per patient		441 (95–5261)	676 (90–4592)	.005
Per 1000 transplant days		22300 (4526–263 072)	32 760 (5027–229 610)	.012

Demographics were assessed via a descriptive statistical analysis. Median or mean values and ranges are provided for continuous variables, while patient numbers and percentages are shown for categorical variables. Unpaired *t* tests or Mann-Whitney *U* tests were conducted for continuous variables, and χ^2^ tests or the Fisher’s exact test were employed for categorical variables. *P* < .05 was considered statistically significant.

Abbreviations: aDDD, adjusted Defined Daily Dose; NPF, neutropenic fever; WHO, World Health Organization.

^a^NPF dosing of 3.375 g every 6 h was selected for piperacillin-tazobactam to serve as a middle ground between the two commonly employed dosing regimens at the institution (3.375 g every 8 h given as prolonged infusion and 4.5 g every 6 h).

^b^Weight-based dosing calculated using a 70-kg patient.

^c^Value reported as mean (range).

^d^Value reported as median (range).

^e^Time from transplant to discharge or death.

## DISCUSSION

To our knowledge, this is the first published study to date evaluating de-escalation of broad-spectrum therapy for neutropenic fever of unidentified origin in adult allo-HSCT recipients who are still neutropenic. While the concept is not entirely new, limited literature exists on early de-escalation in HSCT recipients and the oncology population overall, especially this early in the course of therapy. Our study found that the rate of recurrent fever in the early de-escalation group (cohort 1) was comparable with the rate in those patients who continued on broad-spectrum therapy until neutrophil engraftment (cohort 2). Furthermore, none of the patients in either cohort with recurrent fevers had a subsequent culture-proven bacteremia. The similar baseline and transplant characteristics of the 2 groups (Table 1) serve to validate these findings, as does the lack of difference found in utilization of other potential agents that could contribute to fever or prolonged neutropenia (Table 2). One interesting difference to note is the significantly longer time to neutrophil engraftment in cohort 1 (Table 2). Explanations for this longer duration of neutropenia could be the higher proportions of mismatched unrelated donor transplants and antithymocyte globulin use in this group. Despite a longer duration of neutropenia and potential increased risk of infection, patients in cohort 1 showed no difference from cohort 2 in any of the clinical outcomes measured.

While the primary end point demonstrated that there was no disadvantage to stopping broad-spectrum therapy early, others helped illustrate several potential benefits of utilizing this approach. As expected, cohort 1 had fewer days of broad-spectrum antimicrobial use per patient of both gram-negative and gram-positive broad-spectrum agents, with a significant reduction in the latter (Table 3). Utilization data standardized per 1000 transplant days displayed similar reductions. Cohort 1 had fewer aDDDs for gram-positive and gram-negative broad-spectrum agents. On average, patients in cohort 1 avoided 3.2 days of broad-spectrum therapy, which translated into a reduction in related antimicrobial costs per patient and per 1000 transplant days (Table 3). While a roughly $10 000 difference per 1000 transplant days may not seem significant compared with the overall costs an institution incurs, our small review of 120 HSCT patients alone included a total of 2508 transplant days. This illustrates the possibility of even greater cost savings if such a concept were applied to a larger spectrum of patients.

Some of the difference in antimicrobial utilization between the groups may be attributable to the higher initial use of gram-positive agents at the onset of neutropenic fever in cohort 2. The reason for this difference could not be identified. For patients presenting with neutropenic fever, the NCCN and IDSA guidelines recommend that vancomycin (or an agent with similar gram-positive activity) should be included in the initial regimen if a specific indication like a catheter-related infection, skin or soft-tissue infection, MRSA colonization, pneumonia, or hemodynamic instability exists [8, 9]. In a like manner, an agent with VRE activity (daptomycin, linezolid) should be administed to a VRE-colonized patient. All of these risk factors requiring the addition of extra gram-positive coverage were assessed for each cohort, without any differences found between the 2. There was no difference in rates of MRSA or VRE colonization or the incidence of hemodynamic instability (no difference in qSOFA scores), and patients with any potential sign of infection (catheter-related, skin or soft-tissue, pneumonia) on clinical exam or imaging were excluded from the study completely. Karnofsky Performance Status score, Sorror Comorbidity score, and other baseline characteristics were also similar between the groups. To explore this further, we evaluated patients’ qSOFA beginning at administration of broad-spectrum therapy. This tool from the Third International Consensus Definitions Task Force was found to possess statistically greater predictive validity of in-hospital mortality than SOFA or systemic inflammatory response syndrome in patients with suspected infection outside the ICU [28]. Utilizing its 3 variables of blood pressure, respiratory rate, and mental status provided a better picture of patients’ acute condition and hemodynamic instability over the first 48 hours of broad-spectrum therapy initiation for neutropenic fever. As Table 2 illustrates, there was no difference in mean qSOFA between cohort 1 and cohort 2 in the overall population or the subsets of patients that either did or did not receive initial gram-positive broad-spectrum therapy.

Although one concern with stopping broad-spectrum therapy early may be the possibility of recurrent infection and subsequent need for re-escalation, this was not the case in our study. Whether de-escalating to a prophylactic agent in cohort 1 while the patient was still neutropenic or stopping antibacterial coverage altogether in cohort 2 after the patient had reached neutrophil engraftment, there was no difference in the proportion of patients requiring re-escalation within 72 hours of stopping broad-spectrum therapy. Additionally, examining those who required re-escalation revealed no difference between the groups in time to re-escalation of therapy or subsequent days of broad-spectrum agent utilization. Further, none of the patients with recurrent fevers in either cohort developed a bacteremia or suffered mortality. These low rates of adverse outcomes after early de-escalation are in alignment with the results of several similarly designed studies featured in a recent article by Orasch et al. that argued in support of such practices and the ECIL-4 recommendations [29]. While some variation exists in the trial designs, all demonstrate very low rates of mortality after early de-escalation or discontinuation of broad-spectrum antimicrobials in neutropenic fever treatment, even in those patients with recurrent febrile episodes. On the contrary, a study by Micol et al. found a high rate of bacteremia (2 of 7, 29%) after early antibiotic discontinuation [30]. While concerning, the study design and conclusions had several limitations, including the small population of 7 patients that stopped early, extremely restrictive criteria for discontinuation, failure to acknowledge that the rate of recurrent fever can be frequent in patients whether they remain on broad-spectrum therapy or not, and incorrect assumption that continuing broad-spectrum antibiotics unnecessarily will do no harm to the patient.

Limitations of this study include its retrospective study design, thus the inability to control for all variables that may influence the primary end point in addition to selection bias. This potential selection bias for de-escalation resulted from the Infectious Diseases service’s role as a consult service, which can only make recommendations for early de-escalation. The final decision at our institution rests with the primary transplant team. It is important to note that this study does not demonstrate superiority of early de-escalation of broad-spectrum therapy in allo-HSCT recipients with neutropenic fever of unknown origin, only illustrating that its outcomes appear to be no worse than continuing broad-spectrum therapy until neutrophil engraftment. In our study, patients who were de-escalated from broad-spectrum therapy early did not experience an increased incidence of bacteremias, ICU stay, or mortality. Future prospective studies with larger sample sizes and more robust study design (matched cohorts, randomized controlled trials, etc.) are needed to make any definitive conclusions regarding the superiority of this practice. In addition, any pharmacoeconomic assessments made in this study represent a rough estimate of the potential cost savings as we utilized the overall antimicrobial use data for the analysis. Such assessments were not designed to be the main focus of the article or to draw significant conclusions from, but only to promote the general idea that the implementation of early de-escalation practices could have a positive financial impact at an institution.

Despite its limitations, this study has already impacted current practice at our institution in several ways. Utilization of these data has been implemented into our transplant department’s standard operating procedures. Both autologous HSCT (auto-HSCT) and allo-HSCT patients with neutropenic fever of unknown origin who have received 5 days of broad-spectrum therapy and been afebrile for ≥48 hours are routinely de-escalated back to their original prophylactic agent. Implementation of this protocol has increased the general awareness of the importance of antimicrobial stewardship, not just in the instance of neutropenic fever but for all infections. It has also encouraged the development of a similar study for hematology patients receiving induction therapy, in addition to an even more aggressive approach for HSCT patients in the future—one similar to that recommended by the ECIL guidelines (de-escalating after 3 days of broad-spectrum therapy). General principles from this study could have a great impact on outside practice as well. Early de-escalation of broad-spectrum therapy may promote cost savings on drug purchases and potentially facilitate earlier discharges with patients coming off broad-spectrum intravenous antimicrobials earlier in their stay. More importantly, it can help slow the development of resistant pathogens and infections from fungi and *Clostridium difficile*, all of which become bigger issues as patients remain on broad-spectrum therapy for prolonged periods of time.

## CONCLUSION

In summary, this study demonstrates many advantages of promoting antimicrobial stewardship in allo-HSCT recipients with neutropenic fever. De-escalating to a prophylactic agent after at least 5 days of broad-spectrum therapy and defervescence did not appear to adversely affect the rate of recurrent fever. This practice allowed for a reduction in broad-spectrum antimicrobial utilization, duration, and cost with no difference in key clinical outcomes in comparison with those who continued on such therapy until neutrophil engraftment.

## References

[1] Standiford HC, Chan S, Tripoli M et al. Antimicrobial stewardship at a large tertiary care academic medical center: cost analysis before, during, and after a 7-year program. Infect Control Hosp Epidemiol 2012; 33:338–45. 22418628 10.1086/664909

[2] MacDougall C, Polk RE Antimicrobial stewardship programs in health care systems. Clin Microbiol Rev 2005; 18:638–56. 16223951 10.1128/CMR.18.4.638-656.2005 PMC1265911

[3] ASHP statement of the pharmacist’s role in antimicrobial stewardship and infection prevention and control. Am J Health Syst Pharm 2010; 67:575–577. 20237387 10.2146/sp100001

[4] Nowak MA, Nelson RE, Breidenbach JL et al. Clinical and economic outcomes of a prospective antimicrobial stewardship program. Am J Health Syst Pharm 2012; 69:1500–8. 22899745 10.2146/ajhp110603

[5] Barlam TF, Cosgrove SE, Abbo LM et al. Implementing an Antibiotic Stewardship Program: Guidelines by the Infectious Diseases Society of America and the Society for Healthcare Epidemiology of America. Clin Infect Dis 2016; 62:e51–77. 27080992 10.1093/cid/ciw118 PMC5006285

[6] Centers for Disease Control and Prevention. Core elements of hospital antibiotic stewardship programs Available at: http://www.cdc.gov/getsmart/healthcare/implementation/core-elements.html. Accessed 22 September 2015. 10.1093/cid/ciu542 PMC6521960 25261548

[7] Leung E, Weil DE, Raviglione M, Nakatani H; World Health Organization World Health Day Antimicrobial Resistance Technical Working Group The WHO policy package to combat antimicrobial resistance. Bull World Health Organ 2011; 89:390–2. 21556308 10.2471/BLT.11.088435 PMC3089396

[8] Freifeld AG, Bow EJ, Sepkowitz KA et al. Clinical practice guideline for the use of antimicrobial agents in neutropenic patients with cancer: 2010 update by the Infectious Diseases Society of America. Clin Infect Dis 2011; 52:e56–93. 21258094 10.1093/cid/cir073

[9] National Comprehensive Cancer Network guidelines, version 2.2016 Available at: https://www.nccn.org/professionals/physician_gls/pdf/infections.pdf. Accessed 14 July 2016.

[10] Hodgson-Viden H, Grundy PE, Robinson JL Early discontinuation of intravenous antimicrobial therapy in pediatric oncology patients with febrile neutropenia. BMC Pediatr 2005; 5:10. 15904510 10.1186/1471-2431-5-10 PMC1156908

[11] Chow V, Dorcy KS, Sandhu R et al. Evaluation of early discharge after hospital treatment of neutropenic fever in acute myeloid leukemia (AML). Leuk Res Rep 2013; 2:26–8. 24371771 10.1016/j.lrr.2013.01.001 PMC3850377

[12] Pizzo PA Management of fever in patients with cancer and treatment-induced neutropenia. N Engl J Med 1993; 328:1323–32. 8469254 10.1056/NEJM199305063281808

[13] Gyssens IC, Kern WV, Livermore DM; ECIL-4, a joint venture of EBMT, EORTC, ICHS and ESGICH of ESCMID The role of antibiotic stewardship in limiting antibacterial resistance among hematology patients. Haematologica 2013; 98:1821–5. 24323982 10.3324/haematol.2013.091769 PMC3856956

[14] Averbuch D, Orasch C, Cordonnier C et al. European guidelines for empirical antibacterial therapy for febrile neutropenic patients in the era of growing resistance: summary of the 2011 4th European Conference on Infections in Leukemia. Haematologica 2013; 98:1826–35. 24323983 10.3324/haematol.2013.091025 PMC3856957

[15] Ruhnke M, Böhme A, Buchheidt D et al.; Infectious Diseases Working Party in Haematology and Oncology of the German Society for Haematology and Oncology Diagnosis of invasive fungal infections in hematology and oncology–guidelines from the Infectious Diseases Working Party in Haematology and Oncology of the German Society for Haematology and Oncology (AGIHO). Ann Oncol 2012; 23:823–33. 21948809 10.1093/annonc/mdr407

[16] Lehrnbecher T, Schmidt S, Tramsen L, Klingebiel T Immunotherapy of invasive fungal infection in hematopoietic stem cell transplant recipients. Front Oncol 2013; 3:17. 23404543 10.3389/fonc.2013.00017 PMC3566394

[17] Dubberke ER, Reske KA, Srivastava A et al. Clostridium difficile-associated disease in allogeneic hematopoietic stem-cell transplant recipients: risk associations, protective associations, and outcomes. Clin Transplant 2010; 24:192–8. 19624693 10.1111/j.1399-0012.2009.01035.x PMC3390201

[18] Chakrabarti S, Lees A, Jones SG, Milligan DW Clostridium difficile infection in allogeneic stem cell transplant recipients is associated with severe graft-versus-host disease and non-relapse mortality. Bone Marrow Transplant 2000; 26:871–6. 11081387 10.1038/sj.bmt.1702627

[19] Lehrnbecher T, Stanescu A, Kühl J Short courses of intravenous empirical antibiotic treatment in selected febrile neutropenic children with cancer. Infection 2002; 30:17–21. 11876510 10.1007/s15010-002-2094-1

[20] Cherif H, Björkholm M, Engervall P et al. A prospective, randomized study comparing cefepime and imipenem-cilastatin in the empirical treatment of febrile neutropenia in patients treated for haematological malignancies. Scand J Infect Dis 2004; 36:593–600. 15370671 10.1080/00365540410017590

[21] Kroll AL, Corrigan PA, Patel S et al. Evaluation of empiric antibiotic de-escalation in febrile neutropenia. J Oncol Pharm Pract 2016; 22:696–701. 26227319 10.1177/1078155215597558

[22] Santolaya ME, Villarroel M, Avendaño LF, Cofré J Discontinuation of antimicrobial therapy for febrile, neutropenic children with cancer: a prospective study. Clin Infect Dis 1997; 25:92–7. 9243041 10.1086/514500

[23] Klaassen RJ, Allen U, Doyle JJ Randomized placebo-controlled trial of oral antibiotics in pediatric oncology patients at low-risk with fever and neutropenia. J Pediatr Hematol Oncol 2000; 22:405–11. 11037850 10.1097/00043426-200009000-00004

[24] Agwu AL, Lee CK, Jain SK et al. A World Wide Web-based antimicrobial stewardship program improves efficiency, communication, and user satisfaction and reduces cost in a tertiary care pediatric medical center. Clin Infect Dis 2008; 47:747–53. 18680419 10.1086/591133

[25] Quadri F, Mazer-Amirshahi M, Fox ER et al. Antibacterial drug shortages from 2001 to 2013: implications for clinical practice. Clin Infect Dis 2015; 60:1737–42. 25908680 10.1093/cid/civ201

[26] Center for International Blood & Marrow Transplant Research. Instructions for Post-Transplant Essential Data (Post-TED) Form, version 2 Available at: https://www.cibmtr.org/DataManagement/TrainingReference/Manuals/DataManagement/Documents/post-ted-instruction.pdf. Accessed 19 July 2016.

[27] Norwegian Institute of Public Health. WHO Collaborating Centre for Drug Statistics Methodology: definition and general considerations Available at: http://www.whocc.no/ddd/definition_and_general_considera/. Accessed 4 June 2016.

[28] Seymour CW, Liu VX, Iwashyna TJ et al. Assessment of clinical criteria for sepsis: for the Third International Consensus Definitions for Sepsis and Septic Shock (Sepsis-3). JAMA 2016; 315:762–74. 26903335 10.1001/jama.2016.0288 PMC5433435

[29] Orasch C, Averbuch D, Mikulska M et al.; 4th European Conference on Infections in Leukemia (ECIL-4); joint venture of Infectious Diseases Working Party of the European Group for Blood and Marrow Transplantation (IDWP-EBMT); Infectious Diseases Group of the European Organization for Research and Treatment of Cancer (IDG-EORTC); International Immunocompromised Host Society (ICHS); European Leukemia Net (ELN) and European Study Group on Infections in Immunocompromised Hosts of the European Society for Clinical Microbiology and Infectious Diseases (ESGICH-ESCMID) Discontinuation of empirical antibiotic therapy in neutropenic leukaemia patients with fever of unknown origin is ethical. Clin Microbiol Infect 2015; 21:e25–7. 25658572 10.1016/j.cmi.2014.10.014

[30] Micol JB, Chahine C, Woerther PL et al. Discontinuation of empirical antibiotic therapy in neutropenic acute myeloid leukaemia patients with fever of unknown origin: is it ethical? Clin Microbiol Infect 2014; 20:O453–5. 24313354 10.1111/1469-0691.12445

